# Dengue Infections in Colombia: Epidemiological Trends of a Hyperendemic Country

**DOI:** 10.3390/tropicalmed5040156

**Published:** 2020-10-03

**Authors:** Hernando Gutierrez-Barbosa, Sandra Medina-Moreno, Juan C. Zapata, Joel V. Chua

**Affiliations:** 1 Institute of Human Virology, University of Maryland School of Medicine, Baltimore, MD 21201, USA; hhgutierrez49@gmail.com; 2 Grupo de Virología, Vicerrectoría de Investigaciones, Universidad El Bosque, Bogotá 110121, Colombia; 3 American Gene Technologies International, Rockville, MD 20850, USA; smedinamoreno@americangene.com (S.M.-M.); jzapata@americangene.com (J.C.Z.)

**Keywords:** dengue virus, endemic diseases, epidemiology, Colombia

## Abstract

Dengue is a major public health problem in hyperendemic countries like Colombia, the understanding of the epidemiological trends is important for the development of efficient public health policies. We conducted a systematic review of the epidemiologic data on dengue in Colombia from 1971 to 2020. A total of 375 relevant citations were identified, 36 of which fulfilled the inclusion criteria. The data of dengue and severe dengue cases, infection fatality rate, and serotype distribution were used to understand and identify gaps in the epidemiological knowledge in Colombia. The epidemiology of dengue in this country was characterized by five main outbreaks in 1998, 2002, 2010, 2013, and 2019 with high fatality rates in comparison with the average values reported in the Americas. The case fatality rate of severe dengue exceeded 2% and all four serotypes co-circulate throughout the country with some regional variations. Overall, the behavior of dengue in Colombia is influenced by multiple factors including seasonal temperature variation and socioeconomic conditions. Additionally, the most important barriers in the epidemiological surveillance of dengue may be due to the insufficient notification rate in some regions and the low active search for the circulation of different serotypes.

## 1. Introduction

Dengue is a mosquito-borne disease with an estimated 390 million infections per year in over 125 countries. Roughly 3.9 billion people are currently living in areas at high risk for vector-borne diseases [1], and an increase in the infection rate is expected in endemic areas due to the global expansion of the vector, *Aedes aegypti,* associated with a combination of factors such as climate change, anthropogenic factors, and socioeconomic conditions of the population [2]. Dengue is caused by four closely genetically related types of dengue virus (DENV 1-4) belonging to the *Flaviviridae* [3] and the disease has been classified by the World Health Organization (WHO) according to the clinical presentation as non-severe dengue, dengue with or without warning signs, and severe dengue disease [4].

Trends to global expansion and increasing disease burden, particularly in limited-resource countries, has replaced dengue as a principal public health problem that needs high prioritization in healthcare policy agenda worldwide [2]. In Latin America, the number of cases has dramatically increased in recent years; the last major outbreak was reported in 2019 with 3,140,872 cases [5]. However, in February 2020 the Pan American Health Organization (PAHO) released an epidemiological alert for dengue in the Americas due to an increase of the incidence rate (81.51 cases per 100,000 population), seven times more than reported during the same period in 2019 (11.09 cases per 100,000 population) [6].

Colombia, one of the most affected countries in the American region, has identified dengue as a major public health threat beginning in the 1950s and had identified the first case of dengue hemorrhagic fever (DHF) in 1989 [7,8]. Since then, the incidence and mortality related to DHF have increased annually [9]. The epidemiology of dengue in Colombia has been influenced by increasing human population density, deficiencies in vector control policies enhanced by poor household infrastructure, environmental variations between seasons of drought to high rainfalls, and multiple barriers to accessing health services [10]. These factors made it difficult to predict dengue epidemiologic trends in Colombia. This literature review aims to describe the epidemiology of dengue through analysis of national historical outbreaks, infection fatality rate, severe dengue incidence, serotype distribution, and other relevant epidemiology data allowing us to illustrate the complexity of dengue infection in a country with high geographical diversity.

## 2. Materials and Methods 

We performed a systematic review in epidemiology of dengue infections in Colombia in accordance with the Preferred Reporting Items of Systematic review and Meta-Analyses (PRISMA) guidelines [11]. We aimed to identify all relevant studies regardless of language or publication status in several databases as described below.

### 2.1. Search Strategy and Selection Criteria 

The search of epidemiologic data of dengue in Colombia was carried out in the following databases: Medline (via PubMed), Scientific Electronic Library Online (SciELO), Biomedica (Journal of the Colombian National Institute of Health) and SIVIGILA (Colombian National Epidemiological Surveillance System) online reports. The Medical Subject Headings (MeSH) thesaurus, encompassing the terms “dengue”, “epidemiology”, and “Colombia”. Only articles in English and Spanish published between January 2000 to August 2020 were included; for the early years between 1971 and 2000, the data was extracted from the book “Dengue in Colombia: Epidemiology of Hyperendemic Re-emergence”. No limits by sex, age or ethnicity of the study participants were applied.

We included studies that contained relevant epidemiology data of dengue prevalence, case numbers (of both total and severe cases), infection fatality rate, serotype distribution, serotype frequency. National or regional population epidemiological reports, and population studies (derived from open population-based cohort studies and epidemiological registries) on dengue infections were included. We excluded other types of studies without epidemiological information such as literature reviews, case series, mathematical models, and basic science research.

During the first round of analysis, each title and abstract were independently screened by two reviewers. Any disagreement was resolved by consensus; duplicated articles and those not satisfying the inclusion criteria were excluded. The second round of analysis included full text screen for relevant epidemiological data.

### 2.2. Outcome Measure

Epidemiological data from the included publications were summarized using the data extraction instrument previously reported [12]. Briefly, the data were extracted in the following categories: case number, incidence, disease classification, serotype predominance, serotype presence, dengue mortality rate, and severe dengue mortality rate. Data from literature reviews of previously published peer-reviewed studies were not extracted except for the data in the book “Dengue in Colombia: Epidemiology of Hyperendemic Re-emergence”.

Data were reported as cases per year, incidence density (per 100,000 population), and serotype predominance (percentage per year). Dengue case fatality rate (DCFR) in this review is described as the proportion of presumably infected symptomatic patients dying as a result of dengue virus infection, and severe dengue case fatality rate (SDCFR) described as the proportion of severe cases dying of the disease. Dengue outbreaks are described as the number of cases above the 75th percentile of the mean geometry of cases expected for a specific period of time. The graphics were made in Prisma 8.2.1, Adobe Illustrator, and Adobe Photoshop 2020. 

## 3. Results

**Figure 1:**
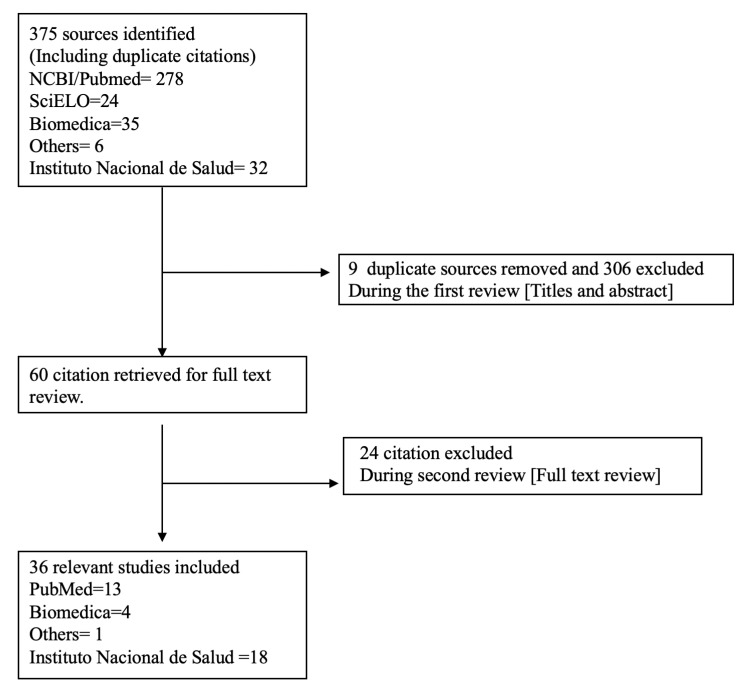
Flowchart of the literature search and included studies.

The search process identified 375 relevant citations. Following the initial duplicate sources removal and Title/Abstract review, 315 citations were excluded. Of these, nine were duplicated, and 306 did not include the epidemiological data relevant to the study or match the scope. Afterward, 60 citations left were full text reviewed, of which 24 citations were excluded since they did not have the relevant epidemiological data or were referred to the mathematical model of the epidemic. Consequently, 36 citations were included for final analysis as shown in Figure 1.

### 3.1. Epidemiology of Dengue in Colombia: Historical Dengue Outbreaks

**Figure 2:**
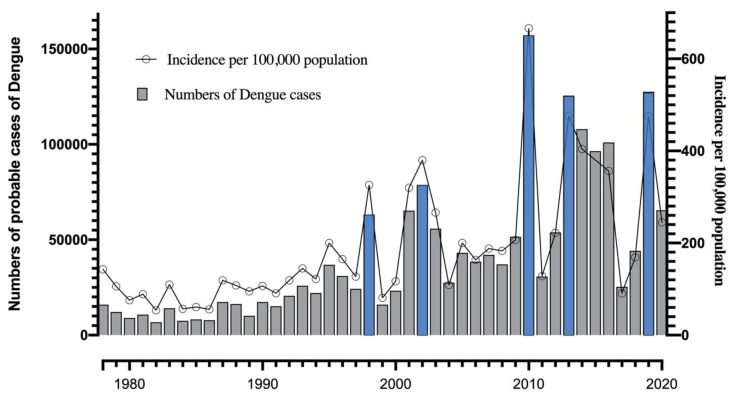
Reported number of probable cases and incidence per 100,000 population of dengue in Colombia between 1978 and the epidemiological week 32 of 2020 [13,14,15,16,17,18,19,20,21,22,23,24,25,26,27,28].

Colombia has shown an increase in reporting and availability of epidemiological data as this country has witnessed multiple epidemics over the years. Despite the efforts made in 1950 by the PAHO programs aimed at eradicating the vectors *Aedes aegypti* and *Aedes albopictus*, the decreased financial support led to the suspension of the program by 1970. Consequently, the incidence of dengue infections significantly increased between 1970 and 1978 with reported cases (and incidence) from 6760 (54 cases for 100,000 habitants) to 36,825 cases (200 cases for 100,000 habitants). After 1978, the number of cases increased gradually up to 24,233 (127 cases for 100,000 habitants) in 1997. Since then five main outbreaks where observed in Colombia as shown in Figure 2 (Blue bars): In 1998 with 63,177 cases (326 cases per 100,000 habitants), 2002 with 78,628 cases (380 cases per 100,000 habitants), 2010 with 157,202 cases (666 cases per 100,000 habitants), 2013 with 125,554 cases (476.2 cases per 100,000 habitants) and 2019 with 127,553 cases (475.4 cases per 100,000 habitants) [6,13,14,15,16]. Of these outbreaks, the highest probable cases and incidence were reported in 2010 [16]. 

### 3.2. Case Fatality Rate of Dengue in Colombia

**Figure 3:**
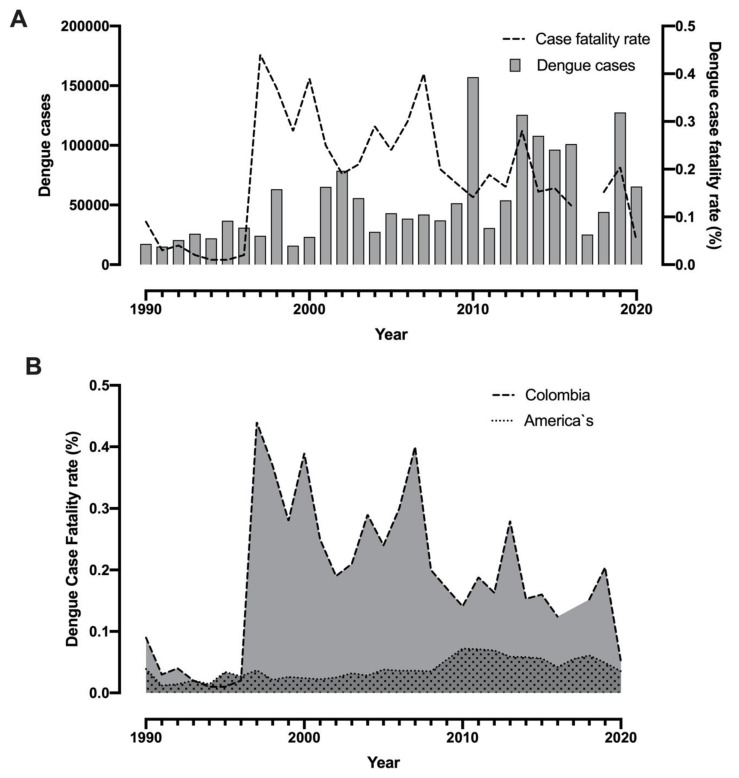
Dengue case fatality rate between 1990 and 2020: Dengue case fatality rate and dengue cases confirmed in Colombia (**A**); Comparison between the Dengue case fatality rate in Colombia and the mean Dengue case fatality rate in the Americas reported by the PAHO (**B**) [5,9,13,14,15,16,17,18,19,20,21,22,23,24,25,26,27,28].

Colombia has a fluctuation in the dengue case fatality rate (DCFR) through the years between 0.01 and 0.44, with six significant peak values. The highest DCFR registered in Colombian history (0.44%) was in 1997 with 106 dengue-related deaths, followed by 2000 (DCFR: 0.39% with 91 Dengue-related deaths), 2004 (CFR: 0.29% with 80 Dengue-related deaths), 2007 (DCFR: 0.4% with 168 Dengue-related deaths), 2013 (DCFR: 0.28% with 352 Dengue-related deaths) and 2019 (DCFR: 0.204% with 261 Dengue-related deaths) [13,14,15,17]. Interestingly, the historical dengue outbreak of 2010 did not have an elevated DCFR (0.141%, 223 Dengue-related deaths) (Figure 3A) [16].

When we compare the mean DCFR reported in Colombia (0.186%), with the mean for the Americas registered by the PAHO database (0.038%), Colombian DCFR is 4.84 times higher than those rates reported in other American countries (Figure 3B), indicating that Colombia has one of the highest values of DCFR in the American region.

### 3.3. Case Fatality Rate of Severe Dengue in Colombia 

**Figure 4:**
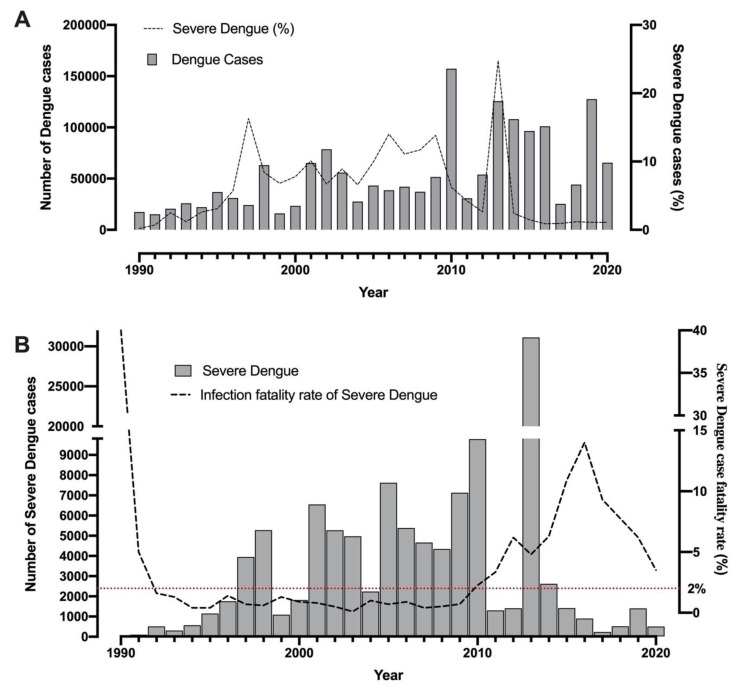
Reported cases and case fatality rate of severe dengue in Colombia: Total dengue cases and their respective percentages classified by severe dengue cases (**A**); severe dengue cases and their respective case fatality rate by year. The red dotted line represents the Colombia government’s goal for the infection fatality rate in severe dengue cases (**B**) [9,13,14,15,16,17,18,19,20,21,22,23,24,25,26,27,28].

The clinical history of dengue in Colombia has had four important peaks of severe dengue cases. The first one registered in 1997 with 107 cases (corresponding to 16.3% of the total dengue cases), followed by 2006 with 5,408 cases (14.0% of the total dengue cases), 2009 with 7,131 cases (13.8% of the total dengue cases), and 2013 with the highest number of severe cases in the period between 1990 and 2020, with 31,113 cases (24.8% of the total dengue cases) (Figure 4A) [1,13,20,29]. When differentiating dengue cases according to their clinical severity, it should be considered that these definitions were made by the WHO in 2009 but were only incorporated in the records and notification sheets at the national level in 2015. The severe dengue case fatality rate (SDCFR) has surpassed the government goal of <2% [30] for the last ten years with the highest values of 6.2% and 14% in the years 2012 and 2016 respectively, with a mean value of 6.68% (Figure 4B) [22,25]. In general, Colombia has a high SDCFR, even more, when considering that WHO, said that the early detection and the proper medical care could lower the values below 1% [31].

### 3.4. Dengue Virus Serotypes Distribution in Colombia 

Dengue, since it was first recognized, has been hyperendemic in Colombia. The first registered serotype was DENV-2 in 1971, followed by DENV-3 in 1975, DENV-1 in 1977, and DENV-4 in 1982 [13]. We found no data with a complete epidemiology surveillance of the frequency of dengue genotypes (e.g., Asian/American genotypes) circulating in the country [8,32,33,34].

**Figure 5:**
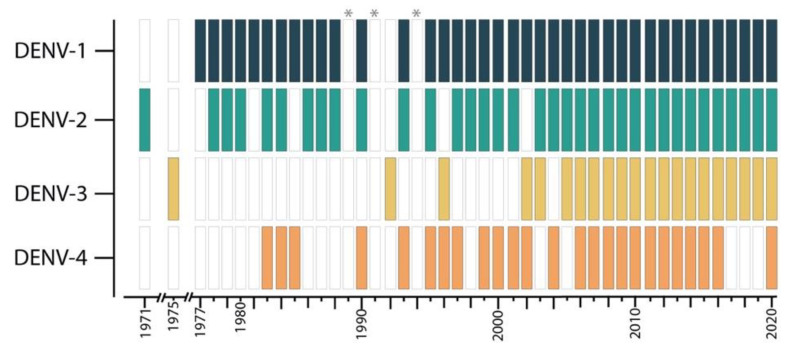
Presence of circulation of Dengue serotypes in Colombia from 1971 to 2020. * No data available [13,35].

Since 1982, the circulation of the different serotypes has fluctuated. However, between 1971 and 2005, DENV-2 and DENV-1 serotypes were more frequently reported. After 2005, a continuous co-circulation of the four serotypes has been described in the country (Figure 5). Though, it is necessary to consider that it was not until 2000 that the sentinel surveillance system started actively monitoring circulating DENV serotypes, there could be some underreporting of data before this year [12].

**Figure 6:**
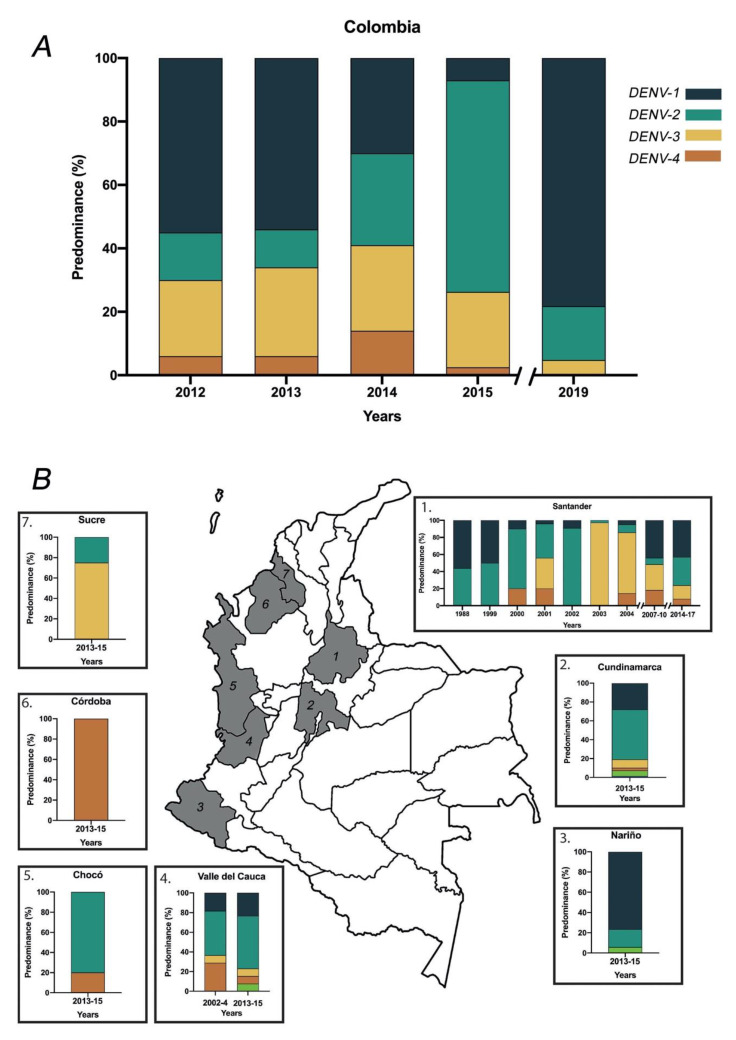
Predominance of the dengue virus by serotypes in Colombia: Predominance rate by serotypes in Colombia by year (**A**); The predominance of dengue virus serotypes by departments in different years (**B**) [14,15,22,23,24,28,35,36,37].

Although the serotypes DENV-1 and DENV-2 are the more predominant serotypes in Colombia, DENV serotypes have a region-dependent fluctuation. The national records between 2014 and 2015 reported a shift of the more predominant serotype, DENV-1 in previous years, towards DENV-2 in this period. However, in 2019, the DENV-1 was reported as the predominant serotype followed by DENV-2. The serotypes with less predominance in the national records were DENV-3, followed by DENV-4 (Figure 6A). Even though the National epidemiological record shows a clear view of the circulation dynamic of the serotypes in Colombia, when we evaluated different departments (a political and administrative division of the country), a fluctuation between serotypes can be appreciated. This is the case of Santander department, where the most predominant serotype has shifted over the years: DENV-1 predominated in 1988−1999, DENV-2 in 2000−2002, DENV-3 in 2003−2004 and again DENV-1 in 2007−2017. Despite the deficit in monitoring the frequencies of serotypes in Colombia, a department dependent serotype proportion was observed (Figure 6B).

### 3.5. Regional Epidemiology of Dengue during the Predominant Outbreaks

**Figure 7:**
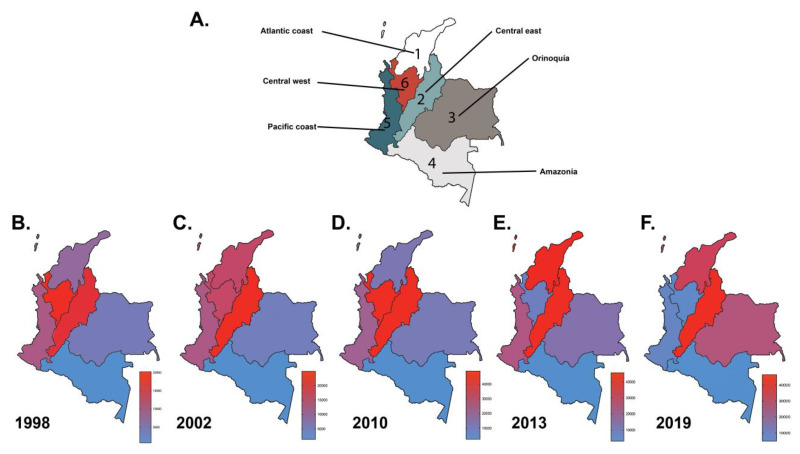
Reported cases of Dengue disease by region through the five most important outbreaks: Division of the Colombian territory by regions (**A**); Heat map of Colombian regions according to Dengue reported cases through the most important outbreaks in 1998 (**B**), 2002 (**C**), 2010 (**D**), 2013 (**E**), and 2019 (**F**) [13,14,15,16].

Colombia is a country that is divided into six regions: Atlantic coast, Central-east, Orinoquía, Amazonía, Pacific coast, Central-west (Figure 7A). There have been regional differences in the epidemiological behavior of dengue in Colombia during the past reported outbreaks. During 1989, 2002, 2010, 2013 and 2019, described as the years with higher dengue cases, the Central-east region contributed with one third of the total dengue cases reported those years, followed by Central-west Atlantic coast, Pacific coast, Orinoquía and Amazonía (Figure 7B–F) [13,14,15,16]. The Central-east region not only has the highest prevalence of dengue through the years but also has reported more frequent severe dengue cases than other regions (Table 1). In the last outbreak, Atlantic coast and Orinoquía region have an increased number of cases compared to previous years. Notably, the Amazonía region has low population density and difficult access to health services, which could contribute to underreporting of dengue cases through the years. 

## 4. Discussion

This study highlights the record numbers of dengue cases in the last 42 years in Colombia. We identified five major dengue outbreaks in 1998, 2002, 2010, 2013, and 2019. Colombia has the highest mean dengue case fatality rate (0.186%), compared to that reported in the American region (0.038%) and higher fatality rate of severe dengue (values of over 2%). Furthermore, a hyperendemic country situation was observed by the co-circulation of different serotypes since 1982, and the constant changes in the dominant serotype by years with a clear department dependent circulation. Finally, we highlight the Central-west and east regions as the most affected during the outbreaks of 1998, 2002, 2010, 2013, and 2019.

The epidemiology of dengue infection is influenced by multiple factors including seasonal temperature variation, socioeconomic conditions, and the evolution of DENV over time. El Niño-Southern Oscitation (ENSO), a phenomenon that resulted in prolonged warm weather, took place in 1997−1998 and 2009−2010 [38], could have explain higher dengue outbreaks during those years. Higher dengue incidence also occurred during years with historical higher temperatures reported in 2002 and 2019 [39]. In addition, the climate variation in temperature and rain season affect the behavior of the mosquito vectors, *Ae. aegypti* and *Ae. albopictus*, leading to an increase of the vector reproduction rate, bite rate, and a shorter incubation period of the mosquito offspring [40,41,42], which translates into an increase in dengue transmission and new cases. Socioeconomic conditions such as those seen when there is lack of adequate housing development and poor public health policy, invariably influences the incidence of dengue. Civil strife results in worsening of socioeconomic well-being of the population, such as those seen in the internal conflict in Colombia between 1996 and 2008. During those years, Colombians migrated from the countryside to the city at a higher rate leading overcrowding in cities with insufficient basic infrastructure and poor housing conditions. As a result, the likelihood of dengue infections increases due to subsequent improper practices for disposal of solid waste, and lack of waste and sewage collection services [9,43,44]. Finally, it is possible that intraserotype strain-specific antigenic variations occur over time in the dengue genetic evolution process, leading to a difference in the binding avidity of the existent antibodies, increasing the infection susceptibility of a person and the odds to progress to severe forms of dengue [45]. We hypothesized that antigenic variation takes place between outbreaks, until eventually the population becomes “immunologically naïve” to newly evolved DENV strains. These antigenic variations over time can explain why a primary antisera developed against a specific serotype may not neutralize all virus strains of the same serotype [3,46], suggesting that people will not only be able to become infected with four different serotypes, but also could be re-infected with the same serotype with a different antigenic strain. The seasonal climate behavior, socioeconomic conditions, and the evolutionary process of dengue virus are not mutually exclusive variables. On the contrary, they complement each other and contribute to the fluctuating behavior of DENV outbreaks.

The large number of cases of severe dengue in Colombia is due to a hyperendemic state that exists in this country. Antibody-dependent enhancement (ADE) occur when people infected with primary DENV infection develop crossreactive DENV antibodies (DENV-Ab) to other serotypes, which wanes over time to sub-neutralizing levels (1:21−1:80), resulting in enhanced secondary heterotypic DENV infection with an increase incidence of severe dengue [47], this mechanism of antibody-mediated enhancement may explain the historic numbers of severe cases in 2013 (125,554 cases). We hypothesize that the 2010 dengue outbreak was the “priming event” that led to a significant proportion of the population exposed and developing cross-reactive DENV-Ab that decayed over the years. When the 2013 dengue outbreak occurred, a large portion of this primed population with sub-neutralizing DENV-Ab was exposed to a new strain-specific antigenic variation or different serotype, resulted in increased incidence of severe dengue. 

In Colombia, the high values of dengue and severe dengue case fatality rates are associated with the quality of medical care, time access to health services, promotion and prevention, poverty, illiteracy, and problems in the clinical practices (mainly related to misdiagnosis and inappropriate treatment) [9]. These limitations can be addressed by public policies that support low-income populations and improving their access to medical care.

The serotype distribution patterns in Colombia are due to genetic flow and evolutionary divergence of the virus. In a population without genetic variation a predominance of one of the genetic variants or serotypes is expected over time [48]. However, Colombia has a significant genetic flow of new viral strains either the same or different serotype from countries such as Venezuela [34], allowing the co-circulation and diversification of the DENV. Moreover, the serotype predominance is region dependent, which implies a regional evolutionary process probably associated with vector populations, immunological characteristics, and geographic barriers that prevent the homogenization of circulating serotypes [49,50,51].

The number of cases reported by region is influenced by the heath care access and the associated number of people who contribute to health insurance due to adequate financial income (contributive system) as opposed to patients who cannot afford health insurance (subsidized system). The access to health service in Colombia, understood as the percentage of people who use medical service when they need it, decreased from 79.1% to 75.5% between 1997 and 2012 [52]. Additionally, in the epidemiology surveillance system the high number of dengue cases are mostly reported by the contributive system while the subsidized system seemed to be underreported [53]. Keeping this in mind, the Central-west and Central-east regions have the largest proportion of people under contributive system with 41% and 54% in 2010, and 56% and 44% in 2013 respectively, while other regions have a low proportion of people in the contributive system [54]. This implies that not all cases of dengue are reported, as well as not all people with the disease have access to the health system, which means that the dengue cases in Colombia are underestimated, and the real numbers are unknown.

Developing a comprehensive dengue surveillance program in Colombia is primarily hampered by insufficient understanding of the dengue epidemiologic trends and behavior and a lack of robust strategies to control the disease. To overcome those challenges, the country must first develop a country-wide comprehensive system of reporting dengue cases. The current state of reporting is limited by the presence of two unequal healthcare systems: the contributive healthcare system were reporting is more accurate and the subsidized healthcare system, which has more patients with poor access to proper medical care resulting in significant underreporting for infectious diseases like dengue. Dengue case registry in the SIVIGILA system captures only symptomatic infections, which presents an important bias in the epidemiologic data as asymptomatic DENV infections play an important role in the viral transmission chain. Lastly, the country must improve the molecular surveillance of dengue; it is necessary to improve the surveillance of the genotypes circulating as well as the relation between them and the incidence or severity of the disease.

## 5. Conclusions

The epidemiology of dengue in Colombia is influenced by three main factors: the seasonal variation in the temperatures affecting the behavior of the vector *Ae. aegypti* and *Ae. albopictus,* the socioeconomic conditions (like insufficient basic infrastructure, housing with poor conditions, improper practices for disposal of solid waste, lack of waste and sewage collection service, and accumulate rainwater that increase the likelihood of DENV infection), and possibly an intraserotype strain-specific antigenic variation (that may explain an increase the risk of suffering severe dengue disease produced by enhancing antibodies). Therefore, the surveillance system of dengue in Colombia has two main challenges: to improve the understanding of Dengue epidemiology behavior and to design efficient strategies to control of disease.

## Figures and Tables

**Table 1:** Dengue and Severe Dengue Cases by Regions in the Five Primary Outbreaks.

Region	Main Outbreaks									
	1998		2002		2010		2013		2019	
	Dengue	Severe Dengue	Dengue	Severe Dengue	Dengue	Severe Dengue	Dengue	Severe Dengue	Dengue	Severe Dengue
Atlantic coast	7072	563	15,332	486	12,627	622	42,167	863	32,341	5178
Central-east	16,137	2333	24,997	1988	49,059	5414	45,726	1007	46,598	504
Central-west	202,117	1100	15,563	490	48,561	850	8842	165	7275	58
Orinoquía	4130	353	4927	67	11,349	685	14,066	177	25,903	185
Amazonía	733	159	1375	103	2519	173	3133	39	4939	40
Pacific coast	9673	707	12,140	1768	20,822	1718	23,226	832	8430	92
Total	57,962	5215	74,334	4902	144,937	9462	137,160	3083	125,486	1397
